# Effectiveness and safety of early medication abortion provided in pharmacies by auxiliary nurse-midwives: A non-inferiority study in Nepal

**DOI:** 10.1371/journal.pone.0191174

**Published:** 2018-01-19

**Authors:** Corinne H. Rocca, Mahesh Puri, Prabhakar Shrestha, Maya Blum, Dev Maharjan, Daniel Grossman, Kiran Regmi, Philip D. Darney, Cynthia C. Harper

**Affiliations:** 1 Bixby Center for Global Reproductive Health, Department of Obstetrics, Gynecology and Reproductive Sciences, School of Medicine, University of California San Francisco, San Francisco, CA, United States of America; 2 Advancing New Standards in Reproductive Health (ANSIRH), Department of Obstetrics, Gynecology and Reproductive Sciences, School of Medicine, University of California San Francisco, Oakland, CA, United States of America; 3 Center for Research on Environment Health & Population Activities (CREHPA), Kusunti, Lalitpur, Kathmandu, Nepal; 4 Ministry of Health, Kathmandu, Nepal; Yale University Yale School of Public Health, UNITED STATES

## Abstract

**Background:**

Expanding access to medication abortion through pharmacies is a promising avenue to reach women with safe and convenient care, yet no pharmacy provision interventions have been evaluated. This observational non-inferiority study investigated the effectiveness and safety of mifepristone-misoprostol medication abortion provided at pharmacies, compared to government-certified public health facilities, by trained auxiliary nurse-midwives in Nepal.

**Methods:**

Auxiliary nurse-midwives were trained to provide medication abortion through twelve pharmacies and public facilities as part of a demonstration project in two districts. Eligible women were ≤63 days pregnant, aged 16–45, and had no medical contraindications. Between 2014–2015, participants (n = 605) obtained 200 mg mifepristone orally and 800 μg misoprostol sublingually or intravaginally 24 hours later, and followed-up 14–21 days later. The primary outcome was complete abortion without manual vacuum aspiration; the secondary outcome was complication requiring treatment. We assessed risk differences by facility type with multivariable logistic mixed-effects regression.

**Results:**

Over 99% of enrolled women completed follow-up (n = 600). Complete abortions occurred in 588 (98·0%) cases, with ten incomplete abortions and two continuing pregnancies. 293/297 (98·7%) pharmacy participants and 295/303 (97·4%) public facility participants had complete abortions, with an adjusted risk difference falling within the pre-specified 5 percentage-point non-inferiority margin (1·5% [-0·8%, 3·8%]). No serious adverse events occurred. Five (1.7%) pharmacy and two (0.7%) public facility participants experienced a complication warranting treatment (aRD, 0.8% [-1.0%-2.7%]).

**Conclusions:**

Early mifepristone-misoprostol abortion was as effective and safe when provided by trained auxiliary nurse-midwives at pharmacies as at government-certified health facilities. Findings support policy expanding provision through registered pharmacies by trained auxiliary nurse-midwives to improve access to safe care.

## Introduction

Unsafe abortion contributes substantially to maternal mortality and morbidity worldwide, accounting for approximately 18% of maternal deaths [1, 2]. Unsafe abortion occurs predominantly in lower-income countries where abortion is illegal or highly restricted, and in remote areas where trained providers and equipment are scarce [3]. In Nepal, where abortion was legalized in 2002 [4], unsafe abortion still accounted for 7% of maternal deaths in 2009, with access to safe and legal care particularly constrained in rural regions [5].

Efforts to improve access to safe abortion have focused on expanding the provider base, as well as decentralizing medication abortion (MA) provision beyond traditional hospital and clinic settings. On the World Health Organization (WHO) Essential Medicines List for developing countries [6], mifepristone and misoprostol abortion is approximately 95% effective in pregnancies under nine weeks, with a gradual decline in efficacy with increasing gestation [7]. The safety and effectiveness of MAB when provided by non-physician clinicians, including nurses and auxiliary nurse midwives (ANMs), has been well-established [8–11], and 2015 WHO guidelines on appropriate health worker roles in abortion care recommend implementing nurse/ANM provision at scale [12]. Accordingly, nurses and ANMs now regularly provide abortion care in Nepal, including in primary-level public health facilities in many districts [13].

Expanding access to MAB through regulated pharmacies may also be a promising avenue to reach women with safe care, though no interventions have been evaluated [14]. In many countries including Nepal, pharmacies are a common first contact point for women seeking abortion and serve as important sources of information and referral [14, 15]. They are more accessible than clinics, particularly in rural areas and for women who have limited autonomy and mobility, and may offer increased privacy compared to public facilities [14, 16, 17]. MAB can be provided without laboratory tests or ultrasounds, and women can safely self-administer misoprostol, complete the abortion, and self-evaluate completion at home [18–20]. In Nepal and elsewhere, however, unskilled and untrained pharmacy workers often provide inaccurate information and dispense unsafe or ineffective methods [21–26], and formative research training pharmacy workers on MAB eligibility and dosing has only been moderately successful [15, 27]. As such the WHO does not currently recommend pharmacist provision [12]. However, other cadres of trained health care providers, including nurses and ANMs, working in pharmacy settings could potentially incorporate MAB provision with clinical protocols into their services, expanding access points for safe services.

Nepal is a leader in innovating access to abortion. Since abortion was decriminalized in Nepal in 2002 [4], access to legal services has expanded to all districts [28, 29]. Abortion is legal for any reason up to 12 weeks’ gestation, and second-trimester procedures, allowed under certain circumstances, became available in 2007. Mifepristone-misoprostol became legally available in 2009, and Nepal is one of the few lower-income countries with national MAB practice guidelines. Provision by nurses and ANMs, who have a tenth grade education and 18 and 24 months’ pre-service formal training, respectively, is permitted. Declines in the severity of abortion complications following legalization have been attributed in part to nurse/ANM service provision as well as the availability of MAB [12, 30]. In a 2013 pharmacy study in two Nepali districts, over 10% of pharmacy workers were trained as nurses or ANMs, and 85% of pharmacies had private spaces for client consultation [31]. Leveraging nurses and ANMs–a trained clinical workforce with a record of safe MAB–to deliver services in pharmacy settings, where women already seek care, could be an important next step toward increasing access to safe abortion.

In this observational demonstration study, we tested the hypothesis that the effectiveness and safety of mifepristone medication abortion provided by trained ANMs through pharmacies would not be inferior to provision through public health facilities that are government-certified to provide MAB. Empirical data on the effectiveness and safety of MAB provision from pharmacies by trained providers do not yet exist but are essential to inform evidence-based policy in Nepal and elsewhere.

## Materials and methods

### Study design and participants

This study was an observational, non-inferiority study comparing outcomes of medication abortion provided by trained auxiliary nurse-midwives in two settings: pharmacies and government-certified public health facilities. Sites were located in both semi-urban and remote regions of the Chitwan and Jhapa districts in the lower Terai region, bordering India. Twelve sites participated: six privately-owned pharmacies and six public health facilities, including four primary health care centers and two health posts. Primary health care centers provide basic health care services, minor surgeries, and aspiration and medication abortion; they are typically staffed by a physician, a few nurses/ANMs, and non-clinical staff. Health posts provide basic health care services and are staffed by a nurse/ANM, a health worker, and non-clinical staff; some are approved to provide medication abortion. Participating pharmacies had to be registered with the Department of Drug Administration, Ministry of Health and Population; have locked storage for data; and have a private space for patient examination, a typical set-up in Nepal [31].

Clinical study procedures were conducted by six ANMs. Each was trained as a skilled birth attendant, had required government training and certification to provide MAB at a public facility, and worked in a pharmacy. MAB outcomes can vary substantially between providers based on experience and tendency to intervene [32]. Because we were evaluating the effect of location of administration, not provider experience or ability, we designed the study such that the same ANM provided services from one pharmacy and one public health facility [31]. Study ANMs were identified in consultation with the District Public Health offices overseeing study sites.

ANMs completed a three-day training on study procedures, ethics and voluntary informed consent, and provision of MAB through the pharmacy, including ensuring confidentiality, storage of clinical case reports, and referrals. At training completion, each participating pharmacy received certification from the Ministry of Health and Population to provide MAB for the experimental demonstration study period. One ANM was relocated during the study; a replacement was trained on-site by a study investigator. Six female research assistants with nursing training were selected and trained to conduct non-clinical data collection.

Women presenting at study sites seeking medication abortion were eligible to participate if they were aged 16–45 and had an established pregnancy of ≤63 days based on pelvic exam performed at the site. Women were excluded if they had already attempted abortion with the same pregnancy, lived outside of Nepal, had any medical contraindications to mifepristone-misoprostol, or were unable to provide consent or complete study visits.

Data were collected October 2014-September 2015. A pilot phase among 57 participants was conducted October-December 2014 to assess feasibility and test instruments. Results of an interim analysis were shared with two University of California, San Francisco (UCSF) obstetrician-gynecologists to determine that complications or incomplete abortions at pharmacy sites were not higher than expected. As pre-specified in the protocol, because no changes to the protocol were required, pilot data were included in analyses.

Ethical approval was obtained from the UCSF Committee on Human Research and the Nepal Health Research Council. The study was endorsed by the Family Health Division, Ministry of Health and Population, Nepal. We followed published guidelines for reporting of non-inferiority trials [33].

### Procedures

For women presenting at all study sites requesting medication abortion, ANMs completed clinical screening for MAB eligibility, as is standard patient care. ANMs recorded last menstrual period and MAB contraindications. They conducted a pelvic exam to assess gestational length and identify signs of ectopic or adnexal mass or infection. Women meeting MAB eligibility criteria (both those enrolling and not enrolling) received MAB counseling, services, and information on when and where to seek care for complications.

Clinical procedures at all sites followed the standard Nepali medication abortion protocol [34], which is consistent with international safe abortion guidelines [18]. Ultrasound and pregnancy confirmation via urine pregnancy test are not part of routine care, but providers could use them or refer women at their discretion. Participants took 200 mg mifepristone orally in front of the provider, followed by 800 μg misoprostol sublingually or intravaginally 24 hours later, usually at home (Medabon®, Sun Pharmaceutical Industries Ltd., Gujarat, India). Due to shortages of Medabon® in study districts, and to eliminate pressure on women to enroll to obtain the drug, the study purchased and provided mifepristone-misoprostol to all women presenting to study sites, regardless of participation. All participants and non-participants were charged 500 Rupees ($5) for MAB care, standard price at public facilities at the time.

Women meeting eligibility criteria provided verbal informed consent. After taking mifepristone, participants completed interviewer-administered baseline questionnaires assessing sociodemographics, pregnancy and contraceptive history, pregnancy intentions, and experiences seeking services.

In the Nepali protocol, patients are provided context-appropriate instructions for when to seek care for a complication or incomplete abortion; follow-up visits are not mandatory [34]. However, to capture study outcome data, participants were asked to return after 14–21 days. At follow-up, ANMs completed a clinical interview about symptoms experienced and any medical care received since enrollment. They conducted a pelvic exam, providing or referring patients for care as needed, including for aspiration abortion for incomplete abortion; additional doses of misoprostol were not provided. Additional follow-up visits were scheduled at the ANM’s discretion until all medical issues were resolved and abortions were complete. Finally, research assistants administered a follow-up questionnaire to capture participant satisfaction. All study procedures were conducted privately and confidentially. Participants received a gift worth 150 Rupees ($1.50) after each interview and 150 Rupees for travel costs at follow-up. Costs of aspiration abortions and treatment for medical care received were covered by the study.

Data were managed by the Center for Research on Environment Health & Population (CREHPA). Research assistants secured data collection instruments at the study sites. Instruments were transferred to the CREHPA offices monthly.

### Outcomes

The primary outcome was successful complete abortion (versus incomplete abortion or continuing pregnancy), without aspiration abortion, within 30 days of mifepristone [9]. Incomplete abortion was defined as the products of conception remaining in the uterus with continued bleeding, enlarged uterus, and open cervix. Continuing pregnancy was when symptoms of pregnancy continued and aspiration abortion was necessary to terminate the pregnancy. Aspiration abortions reported by participants and those determined as necessary by study ANMs at the follow-up visits were included.

The secondary outcome was experience of complications requiring treatment, including any case in which participants sought treatment or ANMs provided or referred for care at a follow-up visit. Serious adverse events, including hemorrhage needing blood transfusion and conditions requiring hospitalization, were captured. Finally, we assessed side effects experienced and satisfaction with services and MAB itself. Data for all outcomes were recorded on follow-up clinical case reports and questionnaires.

### Statistical analysis

This non-inferiority study was designed to assess relative differences in complete abortion among women at pharmacies versus certified public health facilities. We conducted sample size calculations using an expected 97% complete abortions, based on a *Lancet* study of midlevel provider MAB provision [9]. The non-inferiority limit defined -5% as the acceptable difference in successful abortion proportions, based on prior research and feasibility [9, 33]. A sample size of 586 was calculated to be sufficient to establish non-inferiority of pharmacy distribution, with two-sided alpha of 5% and 80% power; an enrollment ratio of 1:1; and increases in the sample of 10% for attrition, 10% to control for sociodemographic differences by arm, and 40% to account for clustering. We based the intraclass correlation coefficient (ICC) on prior research (0.001) [9, 35], increasing it to 0.005 to be conservative. Calculations were replicated varying the expected outcome by 1%, and results did not change substantially.

To assess differences in participant characteristics by arm, we fit a series of bivariable mixed effects models, including random effects for provider and site for clustering. We calculated the proportions of women experiencing complete abortion overall and by arm. To assess non-inferiority of pharmacy MAB as compared to public health facility MAB, we fit a logistic mixed effects model with random provider and site effects. Based on this model, we calculated the crude risk difference by arm and 95% confidence intervals (CI). If the CI fell above -5%, the predetermined non-inferiority margin, we could conclude that pharmacy provision is not inferior to public health facility provision. We repeated mixed effects analyses adjusting for covariables found to differ between arms (education, parity, prior contraceptive use), as well as age and gestational length. We examined complications similarly. We conducted sensitivity analyses, assuming that the five participants who were lost-to-follow-up required aspiration abortions and had complications. Analyses were conducted using Stata v14 (College Station, TX). Results were reported at p≤0.05.

## Results

Participating ANMs were all female and a median of 37 years old (range 30–57). They had spent a median of 16 years (range 6–34) in medical practice and had five years’ experience (range 3–5) providing medication abortion.

**Fig 1:**
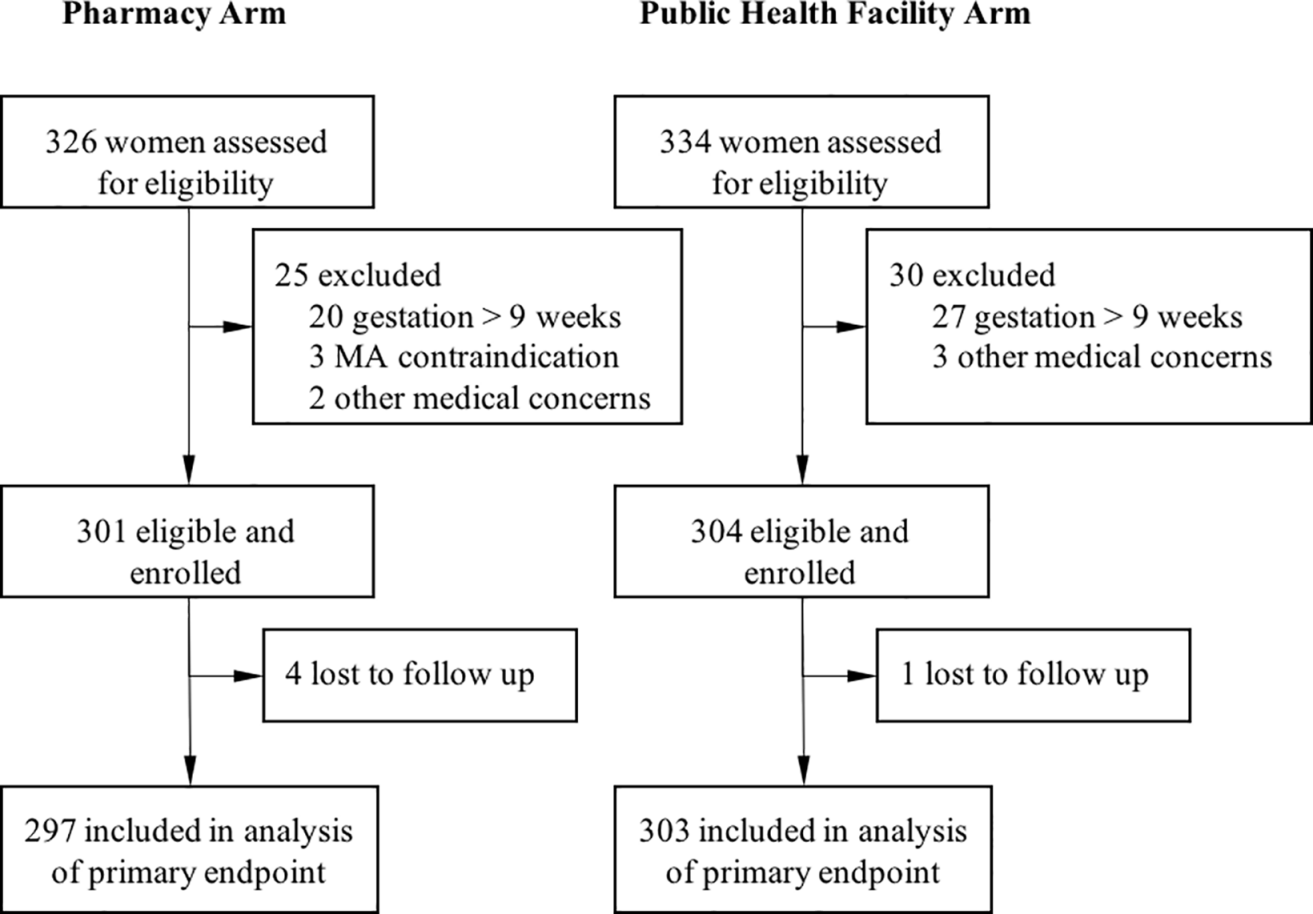
Study profile.

Overall, 660 women presented to study sites seeking MAB during the enrollment period (Fig 1), with 326 presenting at pharmacies and 334 at public health facilities. Of these, 50 did not meet eligibility criteria, with 47 >63 days gestation and three having medical contraindications (two chronic adrenal failure, one bleeding disorder). Two women at pharmacies and three at public health facilities were not enrolled for other reasons (concern of possible uterine infection, anemia, cholelithiasis, hypertension medication, and heart disease). Five of the 605 women enrolled into the study were lost to follow-up after treatment (<1%).

The 605 participants were on average 28 years old (Table 1). Almost all were married (99%), and 94% had children. A majority (61%) worked outside of the home, mostly in farming or livestock. About half had ever used an effective contraceptive method, and 62% wanted no more children. Participants presenting at pharmacies and public health facilities were similar by age, gestational length, prior abortion (33%), and knowledge of abortion legality (50%). However, pharmacy participants had more education (mean 8 vs. 7 years), lower parity (mean 1.7 vs. 2.1), were less likely to have used an effective contraceptive (40% vs. 57%), and were slightly less likely to desire no more children (55% vs. 67%, p = 0.06). Pharmacy participants experienced slightly shorter travel times to the facility than public facility participants (mean 28 vs. 34 minutes, p = 0.06).

**Table 1:** Participant characteristics, by facility type (n = 605).

	Pharmacy	Public Health Facility	p	Total
	(n = 301)	(n = 304)		(n = 605)
	Mean (SD)	Mean (SD)		Mean (SD)
Age (years) (range: 16–44)	27.2 (5.9)	28.0 (6.0)	0.24	27.6 (5.9)
Education (years) (range: 0–17) (n = 604)	7.7 (4.0)	6.8 (4.2)	0.02	7.2 (4.1)
Household assets* (proportion) (range: 0–1) (n = 604)	0.6 (0.2)	0.5 (0.2)	0.12	0.6 (0.2)
Parity (years) (range: 0–7)	1.7 (1.0)	2.1 (1.2)	0.02	1.9 (1.1)
Gestation (days) (range: 35–63)	44.9 (6.0)	44.8 (5.9)	0.57	44.9 (5.9)
Travel time to facility (minutes) (range: 2–240)	28.0 (26.2)	34.2 (36.6)	0.06	31.1 (32.0)
	**n (%)**	**n (%)**		**n (%)**
Married	297 (98.7)	303 (99.7)	0.14	600 (99.2)
Working outside home	192 (63.8)	175 (57.6)	0.24	367 (60.7)
Caste/Ethnicity				
Brahman/Chettri	139 (46.2)	139 (45.7)	0.74	278 (46.0)
Relatively Advantaged Janajatis	17 (5.7)	13 (4.3)		30 (5.0)
Disadvantaged Janajatis/Non-Dalit	119 (39.5)	111 (36.5)		230 (38.0)
Dalit/Untouchable	26 (8.6)	41 (13.5)		67 (11.1)
Parity				
0	23 (7.6)	15 (4.9)	<0.01	38 (6.3)
1	115 (38.2)	79 (26.0)		194 (32.1)
2	117 (38.9)	129 (42.4)		246 (40.7)
3+	46 (15.5)	81 (26.7)		127 (21.2)
Prior abortion	97 (32.2)	101 (33.2)	0.83	198 (32.7)
Future fertility preference				
No more children	167 (55.5)	205 (67.4)	0.06	372 (61.5)
Child > 2 years	116 (38.5)	83 (27.3)		199 (32.9)
Child within 2 years	6 (2.0)	5 (1.6)		11 (1.8)
Don’t know	12 (4.0)	11 (3.6)		23 (3.8)
Ever use of effective contraception	121 (40.2)	172 (56.6)	0.01	293 (48.4)
Know abortion is legal	150 (49.8)	151 (49.7)	0.75	301 (49.8)

* Assessed with 8 items asking whether participants’ households had amenities such as electricity, radio, landline phone, and bicycle.

The proportion of women having complete abortions overall was 98.0%, with 1.7% (n = 10) having an incomplete abortion and 0.3% (n = 2) having a continuing pregnancy (Table 2). By arm, the complete abortion proportions were 98.7% for pharmacy and 97.4% for public health facility, for a crude risk difference of 1.3% [-0.9%, 3.5%]. After adjusting for covariables, the risk difference was 1.5% [-0.8%, 3.8%], with the 95% CI well above the non-inferiority limit of -5%, indicating non-inferiority for abortion effectiveness. All participants with incomplete abortions and continuing pregnancies received manual vacuum aspiration from the study ANM or by referral.

**Table 2:** Medication abortion outcomes (n = 600).

	Pharmacy (n = 297)	Public Health Facility (n = 303)	Risk Difference (95% CI)	Adjusted Risk Difference* (95% CI)
Complete abortion (%)	98.7	97.4	1.3 (-0.9, 3.5)	1.5 (-0.8, 3.8)
Serious adverse event (%)	0	0		
Complication (%)	1.7	0.7	0.9 (-1.0, 2.8)	0.8 (-1.0, 2.7)

* Adjusted models control for age, education, parity, prior contraceptive use, and gestation.

No serious adverse events occurred. Seven participants experienced a complication that warranted treatment based on provider assessment (Table 2). By arm, 1.7% (n = 5) of pharmacy participants and 0.7% (n = 2) of public health facility participants required treatment, mostly antibiotics for fever and potential infection. The risk difference was not statistically significant (aRD = 0.8% [-1.0%, 2.7%]). Participants reported expected side effects, most commonly abdominal cramping (92%), nausea (61%), and chills (42%), with no differences by arm.

Proportions of complete abortions and complications for individual ANMs ranged from 95.6%-99.3% (ICC = 0.001) and 0–3.5%, respectively. Results were consistent assuming the five participants lost-to-follow-up had negative outcomes: complete abortion aRD = 0.4% [-2.2%, 3.1%]; complication aRD = 1.7% [-0.8%, 4.2%].

Acceptability of services was high across sites, with no differences by study arm (Table 3). Satisfaction with MAB as a method was high (64% satisfied, 34% highly satisfied), as was satisfaction with services received (62% satisfied, 36% highly satisfied). Women experiencing an incomplete abortion, continuing pregnancy, or complication were less likely to report satisfaction with MAB (56.3% vs. 99.7%, p<0.001) or services received (78.6% vs. 99.7%, p<0.001). 95% of pharmacy participants and 98% of public health facility participants reported they would prefer to go to a pharmacy and public facility, respectively, if MAB services were needed again (p<0.05).

**Table 3:** Participant experiences with medication abortion, by facility type (n = 600).

	Pharmacy	Public Health Facility	p
	(n = 301)	(n = 304)	
Symptoms experienced:			
Abdominal cramping/pain	92.3	91.4	0.82
Nausea	62.0	60.4	0.68
Chills/shivering	46.1	37.3	0.12
Felt able to manage any symptoms	99.0	97.7	0.23
Satisfaction with MAB as a method			
Highly satisfied	35.0	33.7	0.87
Satisfied	64.0	64.0	
Not satisfied	1.0	2.3	
Satisfaction with services at facility			
Highly satisfied	38.4	35.4	0.82
Satisfied	60.9	63.6	
Not satisfied	0.7	1.0	
Preferred facility for future services if needed			
Pharmacy	94.6	1.7	<0.05*
Public Health Facility	4.7	97.7	
Don’t know/no opinion	0.7	0.7	

* Assessed as preferring to come to the same facility type.

## Discussion

In this observational non-inferiority study, the effectiveness and safety of early mifepristone-misoprostol medication abortion provided by auxiliary nurse-midwives at pharmacies was as effective and safe as compared to when provided by ANMs at government-certified health facilities. Our study showed ANM provision of MAB in both pharmacies (98.7%) and public facilities (97.4%) was as effective as has been demonstrated in other studies using the same regimen (91–99%) [7]. Our findings provide evidence that with minimal additional training, ANMs with previous experience providing MAB can administer services as effectively and safely at a pharmacy as a government-certified facility in a low-resource setting, with high satisfaction across settings.

While nurse and ANM provision expands the abortion provider base, the potential of this trained workforce to reach women is limited by the requirement that services be provided from government-certified public health facilities. Pharmacies are more accessible than clinics, particularly in rural and mountainous areas, and are often the first contact point for women seeking services [14]. The leveraging of pharmacies to expand access to other reproductive health technologies, including injectable and emergency contraceptives, has already been successful in Nepal [36]. Although interventions to train pharmacy workers in harm reduction strategies in Zambia and Nepal have achieved moderate improvement in knowledge and referral practices [15, 27], pharmacists and pharmacy workers, often provide unsafe abortion care [14, 21–26], and the WHO does not currently recommend they provide care. Allowing nurses and ANMs with MAB training to deliver services from pharmacy settings may be the next step in an incremental approach to increasing access to care. Safe pharmacy care from nurses/ANMs may not only mitigate the unsafe or ineffective care women might otherwise receive, but could also expand safe provision beyond hospitals and clinics to a location where women already seek care, thereby reducing unsafe abortion-related morbidity and mortality.

This study has limitations. Public health facility participants returning to the facility for care may have been more likely to receive intervention due to the presence of other clinicians, thereby biasing results in favor of pharmacies; however, incomplete abortions and complications were infrequent in both facility settings in this study. The 14–21 day follow-up period might not have detected delayed complications or ongoing pregnancies. However, study ANM remained in touch with participants until any issues detected were fully resolved, and it is reasonable to believe that most participants who experienced a delayed complication would return to the study facility for care. Our measures of satisfaction did not capture the acceptability of specific aspects of care provided. Finally, given feasibility constraints for a government-approved demonstration project, we were limited to including twelve recruitment sites and six ANMs. We had planned originally for pharmacy providers to rely on self-reported date of last menstrual period for assessment of gestation. Study ANMs, however, were uncomfortable omitting the pelvic exam because they were accustomed to conducting it. Our findings are thus not generalizable to pharmacies without clinical exam space, nor do they apply to unregulated pharmacies or those without ANMs with abortion training. Research should examine whether removing the pelvic exam from the baseline clinical assessment affects abortion effectiveness and safety.

This study is the first to our knowledge to evaluate with a rigorous design and control group the effectiveness of medication abortion provision by trained clinicians in pharmacy settings. Our design isolated the effects of facility environment from the qualifications of providers on medication abortion outcomes. We had high (>99%) follow-up, supporting the internal validity of results. Importantly, this research with ANMs was clinically and ethnically appropriate, conducted in a manner consistent with World Health Organization guidelines, the scientific literature on ANM provision of abortion care, and current clinical practice and law in Nepal. Specifically, the safety and effectiveness of MAB when provided by non-physician clinicians, including nurses and ANMs, has been firmly established in peer-reviewed research [8–11]. The WHO has published evidence-based guidelines delineating the appropriate roles of different cadres of health providers in providing abortion care, recommending the implementation of nurse and ANM provision of first-trimester MAB care at scale as a safe and effective strategy for increasing access to safe pregnancy termination [12]. ANM provision of first-trimester MAB care is legal in Nepal for nurses and ANMs trained as skilled birth attendants and who have completed government training and certification. ANMs currently provide medication abortion care throughout Nepal [13, 29].

### Conclusion

Despite improvements in access to safe abortion care in Nepal, women continue to face obstacles to safe abortion and to seek care from unskilled providers, with access to safe care particularly constrained in rural regions [5]. This study provides data supporting the expansion of medication abortion services to registered pharmacy settings when care is provided by an appropriately trained ANM. Future research should build on these findings by investigating ways to safely implement pharmacy ANM provision at scale and monitor quality of care provided.
